# Supplementary material for: Qualitative Exploration of Anesthesia Providers’ Perceptions Regarding Philips Visual Patient Avatar in Clinical Practice
Source: Bioengineering (Basel). 2024 Mar 27;11(4):323. doi: 10.3390/bioengineering11040323 (PMC11048149; doi:10.3390/bioengineering11040323)
Supplement: Supplementary file 1 [file bioengineering-11-00323-s001.zip › Supplementary Figure S1.pdf]

IntelliVue Patient Monitor
Visual Patient Avatar Quick Guide

Release P.0 onwards

Read the Instructions for Use before monitoring patients.

Monitoring with Visual Patient

The Visual Patient Avatar is an animated virtual model of a monitored patient. The avatar indicates a dynamic change in a vital sign parameter, but the changes in pace, color, or filling of an animation do not necessarily correlate with the pace of changes in measurement values.

Changes in the visualization of the Visual Patient Avatar are only defined by the thresholds set for the individual parameters. The alarm limits for these parameters do not define changes.

Visualizations

Heart Rate

The heart is colored red, and the yellow arrow inside the heart moves downward. The pace of the arrow can be moderate, slow, or fast, depending on the patient's state.

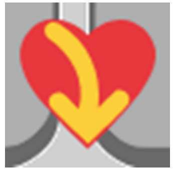	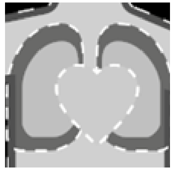
Moderate, slow, or fast pace.	Visualization off/unavailable.

Pulse Rate

The avatar's body pulsates. The frequency changes to very high or very low once the respective thresholds are crossed.

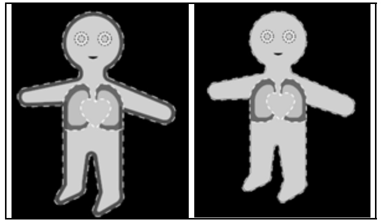

ST Analysis

A small dark shape is displayed within the heart, depending on the location of the ST segment elevation.

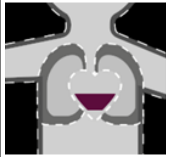	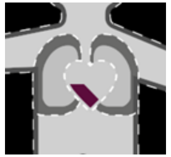
Inferior	Posterior
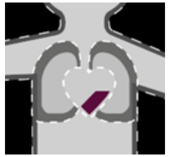	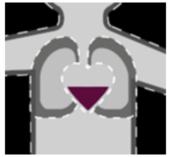
Anterior	Multiple

Arterial Pressure

The outline of the avatar expands up to a certain point, depending on the patient's state.

Within thresholds:	Below lower threshold:	Above upper threshold:
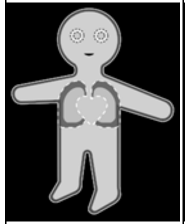	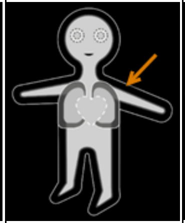	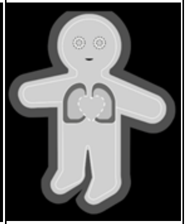
The outline expands to the body boundary.	The outline does not expand to the body boundary.	The outline expands beyond the body boundary.

If the visualization is off, or unavailable, a dotted line appears around the body.

Central Venous Pressure

A vein, indicating the vena cava, is displayed next to the heart.

Within thresholds:	Below lower threshold:	Above upper threshold:
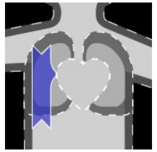	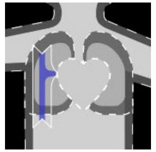	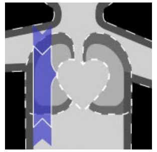
The vein is fully filled.	The vein is partially filled.	The vein is filled beyond its boundaries.

Oxygen Saturation

The color of the avatar represents the oxygen saturation.

Within thresholds:	Below lower threshold:	Above upper threshold:
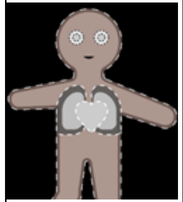	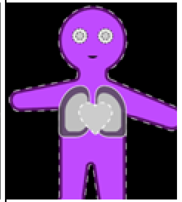	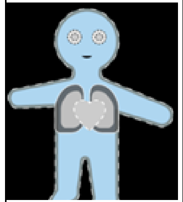
The color is light brown.	The color is violet.	The color is light blue.

If the visualization is off, or unavailable, the color of the avatar is gray.

End-tidal CO_2

End-tidal CO_2 is visualized with an air bubble coming out of the avatar's mouth.

Within thresholds:	Below lower threshold:	Above upper threshold:
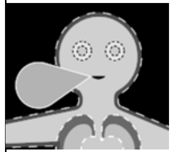	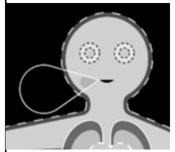	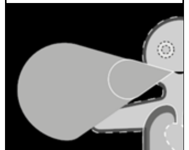
The air bubble is completely filled.	The air bubble is partially filled.	The air bubble is filled beyond its outline.

Respiration Rate

The lungs are displayed with a breathing animation. The breathing can have moderate frequency, low frequency, or high frequency, depending on the patient's state.

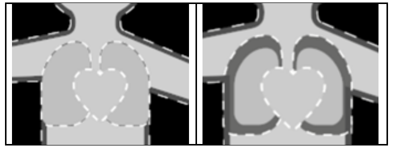

453665023571

CE 0123

Body Temperature
Symbols next to the avatar show when the thresholds are crossed.

Below lower threshold:	Above upper threshold:
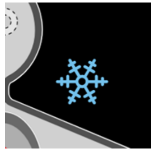	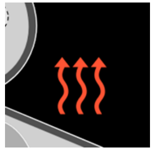
Snowflakes are displayed.	Heat waves are displayed.

Cardiac Output
An aorta is shown with red dots, indicating red blood cells that are ejected into the aorta, according to the patient's cardiac output.

Within thresholds:	Below lower threshold:	Above upper threshold:
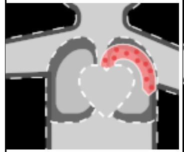	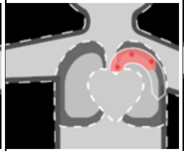	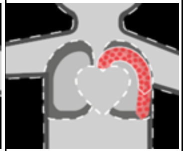
Aorta is filled completely.	Aorta is partially filled.	Aorta is filled beyond its boundaries.

Brain Activity (BIS or PSI)
The avatar's eyes visualize the brain activity.

Below lower threshold:	Above upper threshold:
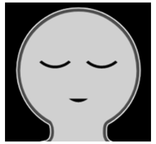	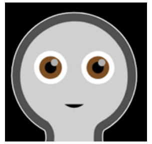
The eyes are closed.	The eyes are open.

If the visualization is off, or unavailable, a dotted line appears around the eyes.

Relaxation (NMT - TOF ratio)
The level of relaxation is represented by the posture of the avatar.

Within thresholds:	Below lower threshold:
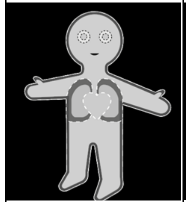	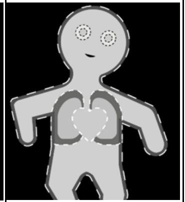
The avatar is displayed with thumbs up.	The limbs are displayed as hanging.

Airway Pressure
The trachea of the avatar shows a filling. The filling is animated with the frequency of the respiration rate.

Within thresholds:	Below lower threshold:	Above upper threshold:
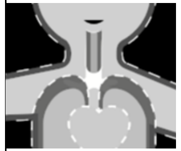	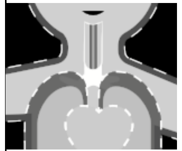	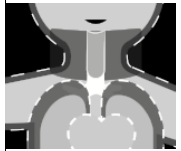
The trachea is filled.	The filling shrinks.	The filling expands.

FiO_2
The color of the lungs and, if airway pressure is available, the color of the trachea, change according to the state of the patient.

Within thresholds:	Below lower threshold:	Above upper threshold:
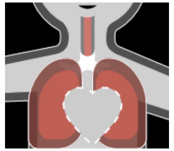	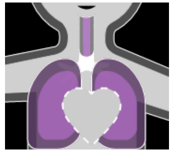	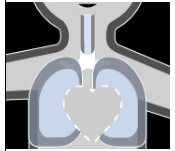
The lungs and the trachea are red.	The lungs and the trachea are pink.	The lungs and the trachea are blue.

Tidal Volume
The lungs are filled by an animation. This filling expands or shrinks, depending on the patient's state.

Within thresholds:	Below lower threshold:	Above upper threshold:
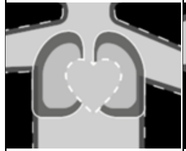	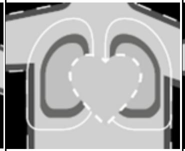	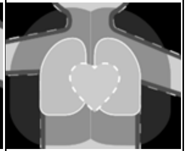
The lungs are filled by an animation.	The filling shrinks.	The filling expands.

If the visualization is off, or unavailable, a dotted line appears around the lungs.

Troubleshooting

What can I do if a parameter is unavailable for Visual Patient Avatar?
If a parameter is unavailable, the display shows the "unavailable" status for this parameter. This is indicated by gray color, dotted lines, or no representation at all.
Troubleshoot the issue as follows:

- Make sure that the Visualization state of the measurement is turned on in the setup menu.
- Check that the measurement is not in a technical alarm condition.
- Check that you selected the right source.
- Check that the measurement is not deactivated, for example due to a label conflict.

The NBP measurement is an aperiodic measurement. How long does the avatar visualize the blood pressure?
The Visual Patient shows the NBP in the same manner as the invasive blood pressure. The configured aging time in the monitor defines how long the avatar visualizes the patient's blood pressure. The factory default is 10 minutes.

What can I do If the preconfigured thresholds are not suitable for my patient?
You can switch the visualization for the respective parameter off.

Obtaining further information

Your monitor Instructions for Use (IfU) contain important safety information. This Quick Guide is not intended as a replacement for the IfU; you must be familiar with the information in the IfU before you begin monitoring patients.

Published in Germany, MAY 2022
©Copyright 2022. Koninklijke Philips N.V.
All Rights Reserved
