# Supplementary material for: Qualitative Exploration of Anesthesia Providers’ Perceptions Regarding Philips Visual Patient Avatar in Clinical Practice
Source: Bioengineering (Basel). 2024 Mar 27;11(4):323. doi: 10.3390/bioengineering11040323 (PMC11048149; doi:10.3390/bioengineering11040323)
Supplement: Supplementary file 1 [file bioengineering-11-00323-s001.zip › Supplementary File S1.pdf]

Supplementary File S1: Translated survey invitation

Dear team

A few months after the introduction of the Philips Visual Patient Avatar into clinical practice, we would like to ask you for your opinion on this innovation.

Your feedback will help us to identify the positive aspects of the technology, recognise potential avenues for improvement and contribute to the further development of this and other situational awareness based technologies.

To gather your opinions, we have prepared a short survey.

Link to the survey (duration approx. 3 minutes):

XXX

Best regards and thank you for your feedback!
