# Supplementary material for: Qualitative Exploration of Anesthesia Providers’ Perceptions Regarding Philips Visual Patient Avatar in Clinical Practice
Source: Bioengineering (Basel). 2024 Mar 27;11(4):323. doi: 10.3390/bioengineering11040323 (PMC11048149; doi:10.3390/bioengineering11040323)
Supplement: Supplementary file 1 [file bioengineering-11-00323-s001.zip › Supplementary Table S1.pdf]

Supplementary Table S1: Translated survey questions

Visual Patient Real-Life Introduction: a Qualitative Study

Part 1: Open questions about Visual Patient

1. What do you like about Visual Patient, having already worked with the technology?
2. Where do you see potential for improvement in Visual Patient?
3. Is the existing teaching material sufficient? Do you miss any training/learning material for using Visual Patient?

Part 2: Demographics

1. Please enter your age: ....
2. Please specify gender:<br>a) Female;<br>b) Male;<br>c) Other Gender Identity.
3. Please indicate your position:<br>a) Nurse anesthetist in training;<br>b) Certified* Nurse Anesthetist;<br>c) Resident 1st-2nd year;<br>d) Resident 3rd-5th year;<br>e) Intern with > 5 years of experience;<br>f) Senior Physician;
4. Please indicate your anesthesia experience (in years): ....
5. Have you viewed any Visual Patient educational materials? If so, which ones?<br>a) Quick Start Guide pdf (Intranet);<br>b) Introductory video (Intranet);<br>c) Both;<br>d) I have not viewed any training materials yet.
