# Supplementary material for: Qualitative Exploration of Anesthesia Providers’ Perceptions Regarding Philips Visual Patient Avatar in Clinical Practice
Source: Bioengineering (Basel). 2024 Mar 27;11(4):323. doi: 10.3390/bioengineering11040323 (PMC11048149; doi:10.3390/bioengineering11040323)
Supplement: Supplementary file 1 [file bioengineering-11-00323-s001.zip › Supplementary Table S2.pdf]

Supplementary Table S2: Translated statements from the responses of the online survey, categorized as perceived positive features, areas for improvement, and non-codable statements.

Statements about perceived positive features		
**Participant**	**Statement Number**	**Statement**
1	1	Visual Patient is amusing
7	1	[I like the] clarity provided by Visual Patient
	2	ability to assess the situation, even from a distance, when numbers are no longer legible
9	1	Good visibility of alarms, even from a greater distance (although not relevant in anesthesia practice)
11	1	The rapid visible changes [are beneficial]
12	1	Visualizing temperature
13	1	Good color contrast
	2	[I like the] Snowflakes
15	1	Problems are quickly and easily identified
16	1	Good overview
	2	[Visual Patient gives me] the ability to quickly identify all problems in a dynamic situation
17	1	All vital parameters are visually presented
	2	and easily recognizable
18	1	The purple discoloration helps me particularly with saturation at 94
19	1	A good addition to the numerical display
21	1	I find the cyanosis detection very good
24	1	[Visual Patient] provides a quick overview, especially when things get hectic, without triggering alarms
25	1	Useful supplementary tool
26	1	Clear structure
	2	easily visible
27	1	I notice when the Visual Patient shows a low temperature or poor saturation because the visual appearance changes significantly
30	1	Clear visual support for identifying the underlying problem when there is a deviation in "one" of our measurement parameters
32	2	I like the intuitive understanding of my patient's circulation
33	1	[I like the] easier identification of ST changes
34	1	Heartrate [is particularly easy to identify]
	2	Hypoxemia [is particularly easy to identify]
	3	hypertension [is] particularly easy to identify
35	1	Pathologies can be easily detected from a distance
37	1	Quick overview of various parameters
38	1	Fast and good situation awareness
	2	Problems are noticed more quickly
39	1	Vital parameters at a glance
41	1	Funny additional information that usually works

42	1	You get used to it very quickly
44	1	Provides a general overview of whether everything is within the normal range
45	1	A tool that can be helpful, especially for beginners
47	1	Provides a quicker overview alongside the numbers. First glance at the avatar, then the numbers
50	1	Rapid identification of problems
52	1	Simplicity
53	1	It summarizes the most important data at a glance
54	1	[Good] visualization of the patient
	2	The purple color immediately catches the eye, indicating a drop in saturation
55	1	[I like] the temperature display
56	1	Simplicity
58	1	Good size (doesn't dominate the screen)
59	1	I feel that my awareness of changes in vital parameters is higher
60	1	Even non-IFA personnel notice changes, leading to more interdisciplinary discussions about vital parameters
61	1	On the first glance, a lot of information can already be inferred
	2	The different colors and symbols are easily distinguishable and provide good indicators
62	1	Temperature display [is good]
63	1	The visual representation is intuitive and easy to understand

Perceived areas of improvement		
**Participant**	**Statement Number**	**Statement**
1	2	When displaying multiple parameters (in ICU patients, neurosurgery, cardiac surgery), it becomes bothersome
	3	limited space on the monitor
	5	[should be] smaller
2	2	too much blinking
	3	It [should] react to the user-set alarm thresholds and [should not be] pre-set
3	1	In my opinion, it does not simplify everyday tasks
	5	[User-set] alarm thresholds should be automatically adopted
	7	Frequent incorrect representation of vital parameters (e.g., lung moving in sync with pulse at frequencies of 90/min)
5	2	It takes up too much space on the monitor
	4	individual limits cannot be set
6	1	In my view, it has little utility. With several years of professional experience, I focus on the values, not the avatar
	2	For example, during a stroke, the avatar blinks even though high blood pressure thresholds are intentionally set. This only confuses the user
	3	The user-set thresholds should be considered as the norm since they are intentionally chosen

7	3	A larger portion of the split monitor should be dedicated to curves and measurements that are incorporated into the avatar
8	2	Nobody pays attention to the VP
9	3	The reduction of the remaining curves is a major point of criticism
	4	Adjustable alarm thresholds, or automatic adoption of monitor alarm thresholds
10	2	Integration of trends (e.g., temperature)
11	2	in the settings, some do not correspond to the presets
	3	some reactions are confusing
12	2	The alarm thresholds must correspond to the alarm thresholds set for normal vital parameter settings. Otherwise, there are too many alarms
13	3	Ability to independently adjust limits would be great, so that values that are too high or too low could be adjusted for the individual patient
14	3	For SpO2, you may want to enter changeable values so that a COPD patient is not displayed as purple all the time
15	2	Adjust the reference values for O2 saturation - 95% is quite high
16	3	Link the limits of the VP to the alarm thresholds, possibly with a small offset
17	3	[I would like to see] more gradations, for example, in cases of hyper- or hypotension or SpO2 (not just good or bad or more than 2 colors
18	2	The cyanosis could be further differentiated in color
19	2	Ability to set limits for Visual Patient oneself. BIS, saturation... triggers alarms too early
20	1	Currently, it only serves as a screensaver when the X3 is on the monitor or during transport
	2	For me, it is a waste of technical resources since the technology is not designed for this amount of data
	4	Furthermore, the technology is far from being advanced enough for the avatar to automatically adjust to alarm thresholds
	5	[I would prefer] automatic adjustment to the alarm thresholds, regardless of how they are set
21	2	It would be helpful if the color could be further differentiated. Similarly, CO2 could be color-coded for hyper- or hypocapnia
22	1	No added value for my work; I tend to ignore it.
	2	Alarm thresholds should apply according to the set limits.
23	2	Setting of threshold values
24	2	Adjustable limits/trends
25	2	Individualization of thresholds
26	3	Age-specific alarm thresholds
27	2	I must say that I tend to ignore the Visual Patient; I rely on numerical values and curves as they are more informative
28	1	Unfortunately, no advantages are apparent.

	2	Not applicable for highly specialized anesthesia.
29	1	The ability to turn it off
	2	The ability to turn it off with just one button press
	3	If it cannot be turned off, it would be advantageous to enter custom alarm thresholds
30	2	If multiple parameters deviate near the limits, the overview is lost because too many stimuli are displayed at the same time
	3	This requires disciplined adjustment of the thresholds. I would like an adapted option with situation-adaptive thresholds to maintain the overview
31	2	Too restless
	3	no customization options (alarm thresholds, acknowledgment, etc.)
32	3	The alarm thresholds must be adjustable (e.g., for a patient with low SpO2, but it can be tolerated)
	4	The current visual representation during tachycardia is too hectic. I cannot work with the VP for tachycardic patients. It is like a stroboscope. I understand that you deliberately did not choose the actual frequency for display. Please reconsider this. You might be hindering yourselves with this dogma
	5	The graphical representation of the VP needs to be more professional. Patients often find it strange and point this out. Perhaps a project with design students could help. Currently, it still looks like a homemade gingerbread figure and not quite professional (Sorry...).
	6	Why not have both a male and female VP? That would be cool, but it's not really a criticism. Just an idea
33	2	Adjustable alarm thresholds
34	4	[It is difficult to perceive the information] when multiple problems occur simultaneously
35	2	Dedicated function, particularly regarding hemodynamics
36	1	Deactivation
	2	Not suitable for anesthesia professionals; perhaps more suitable for a different target audience without extensive training
37	2	The pulsation of the Visual Patient indicates whether the pulse is within the normal range. However, the frequency of pulsation does not match the heartbeat heard via SpO2. This is very confusing because what is seen does not correspond to what is heard
38	3	[I would like the] inclusion of pacemaker function (as in a regular EKG)
39	2	Make alarm thresholds customizable
40	1	To be honest, I don't pay attention to it
	2	Implement alarms with less binary responses, e.g., BIS eyes open when EEG fluctuates slightly above 60

41	2	Better gradation of limits. For example, 36 degrees is too cold, and 36.1 is a normal temperature, not really informative
43	1	Currently, I don't pay attention to the avatar
	2	Not evaluable since I don't pay attention to the avatar
44	2	Too many pieces of information on too little space
	3	No trend visible
	4	No adjustment of normal values possible
45	2	Unable to set limits on the doll
	3	very hectic in children (tachycardia)
46	1	Not very helpful
	2	As long as the parameters cannot be adjusted specifically for each patient, the displayed deviations from normal parameters are unusable
47	2	Personally, I am still undecided whether it would be better to base the heart rate on the actual rate or some form of clustering. Perhaps multiple levels <40, 40-60, 60-100, 100-140, >140
	3	In general, are there already too many parameters displayed in VP and should it be reduced to the basics?
48	2	Remove the avatar from the screen
49	2	[Requires improvement in] TOF visualization
51	1	Most of the time, I focus on the "old" curves
	2	SpO2 turns deep blue from 95%
53	2	Adjustment of saturation limit to a minimum of 92%
54	3	The heart could also have a noticeable color, like green
55	2	Synchronization of pulse sounds with the pulsatile blood pressure display of the VP
57	2	The eyes are spooky
	4	does not provide the expected information
58	2	[Area of improvement:] alarm thresholds
59	2	Representation. ST-segment changes (with indicated "ischemia") are technically invalid when, for example, "Auto Size" is selected, as the "ischemia" suddenly disappears
62	2	Linking with alarm thresholds
63	2	Alarm thresholds need to be adjustable. A defined default is useful; for example, in thoracic surgery, a "blue" patient with SpO2 92% is often more confusing than helpful

**Non-codable statements**		
**Participant**	**Statement Number**	**Statement**
1	4	rather distracting
2	1	Rather unnecessary
3	2	on the contrary, it distracts from vital parameters
	3	frequently displays them incorrectly

	4	It should be possible to turn it on and off based on user preference
	6	Automatic detection when monitoring is not connected (e.g., BIS) to avoid false readings
4	1	To hide it
5	1	Patients feel entertained
	3	cannot be hidden
8	1	The best option is to stop working with it
9	2	Overall, I have a negative impression of the technology
10	1	Helplessly cute
14	1	Good
	2	In addition, [I would like] an early warning system, for example, for temperature
20	3	the monitors often have problems
	6	The technology should be designed for the amount of data and should not lag behind for several years
23	1	Distracts from the raw data
31	1	Minimal [benefits]
32	1	First and foremost, I am a fan of VP. It is really great
42	2	Legend
48	1	No advantages apparent; it is a distracting factor on the screen, diverting attention from the essentials
49	1	Pediatrics
57	1	It's okay
	3	Currently often too confusing to look at
